# Case report: Dupilumab-induced linear psoriasis: a rare presentation

**DOI:** 10.3389/fmed.2024.1527257

**Published:** 2025-01-27

**Authors:** Wen Sun, Kexin Wang, Yuekang Ren, Huajie Yuan, Xiaoqing Lang, Shuping Guo, Hongye Liu

**Affiliations:** ^1^The First Clinical Medical College, Shanxi Medical University, Taiyuan, Shanxi, China; ^2^Department of Dermatology, First Hospital of Shanxi Medical University, Taiyuan, Shanxi, China

**Keywords:** dupilumab, linear psoriasis, prurigo nodularis, case report, chimeric gene

## Abstract

A 42 year-old male with prurigo nodularis treated with dupilumab showed a case of linear psoriasis, highlighting the potential of dupilumab to induce immune shift and chimeric gene expression.

## Introduction

Linear psoriasis is a rare type of psoriasis characterized by a linear distribution of psoriatic lesions along the Blaschko’s line. Although the pathogenesis of linear psoriasis is not well-understood, the involvement of genetic chimerism has been proposed as a major driver of its occurrence. Prurigo nodularis (PN) is a chronic inflammatory skin disease which presents as a persistent solitary pruritic hard papules and nodules. Dupilumab has been approved by the Food and Drug Administration (FDA) for the treatment of adults with PN. In recent years, a subset of patients with PN was reported to experience immune shift following dupilumab treatment, resulting in psoriatic lesions (1, 2). A case of linear psoriasis in a patient with PN treated with dupilumab was presented. This case highlights the potential of dupilumab to induce not only immune shift but also chimeric gene expression.

## Case reports

**FIGURE 1:**
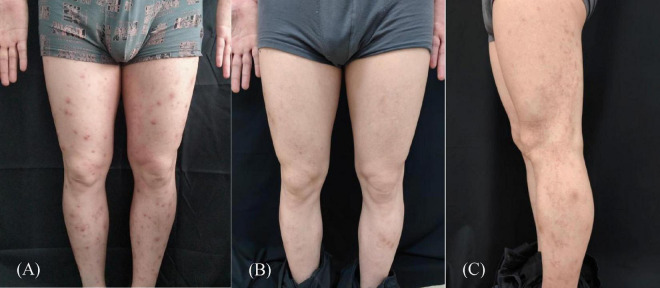
**(A)** Pruritic erythema and scattered nodules on the patient’s both lower limbs; **(B)** after 3 months of treatment, the pruritic erythema and scattered nodules disappeared completely; **(C)** well-defined erythematous squamous papules and plaques on the posterior surface of the left thigh that were linearly arranged along the Blaschko line and extended to the ankle joint.

**FIGURE 2:**
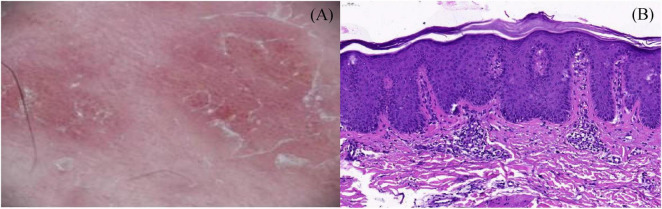
**(A)** Dermoscopic evaluation detected whitish scales and regularly distributed simple red loops, twisted red loops, and globules; **(B)** skin biopsy was performed, and histopathology showed hyperkeratosis and parakeratosis, regular acanthosis, thin granular layer, dilated blood vessels at the tip of papillary dermis and perivascular infiltration of lymphocytes (H&E, 200×).

A 42 year-old male visited our department due to pruritic erythema and scattered nodules on his trunk and limbs for more than 10 years. Routine blood test, thyroid function, liver and kidney function tests, HIV, syphilis test, and the serum total IgE level found no abnormal results. The patient denied a personal and family history of atopic diseases and psoriasis. Based on the above background, a diagnosis of prurigo nodularis (PN) was made. Despite treatment with oral antihistamines, gabapentin, thalidomide, topical glucocorticoid, the patient’s condition did not improve. Three years ago, he was treated with dupilumab. After 4 months of use, erythematous scaly papules and plaques in a Blaschkoid distribution appeared on the right lower limb. Topical treatment with fluticasone propionate cream and calcipotriol ointment for 9 weeks completely resolved the right lower limb rash. Moreover, systemic erythema and nodules were cleared, and pruritus exhibited significant improvement, and hence, dupilumab treatment was gradually discontinued. The pruritic erythema and scattered nodules recurred 1 year ago. The patient was put on dupilumab treatment again at an initial dose of 600 mg iH, which was

reduced to 300 mg iH every 2 weeks. After 3 months of treatment, the original lesions disappeared completely, but pruritic linear lesions appeared in the left lower limb. Further examination revealed well-defined erythematous squamous papules and plaques on the posterior surface of the left thigh that were linearly arranged along the Blaschko’s line and extended to the ankle joint (Figure 1). Dermoscopic evaluation detected whitish scales and regularly distributed simple red loops, twisted red loops, and globules (Figure 2A). Skin biopsy was performed, and histopathology showed hyperkeratosis and parakeratosis, regular acanthosis, thin granular layer, dilated blood vessels at the tip of papillary dermis and perivascular infiltration of lymphocytes (Figure 2B). Therefore, the diagnosis of linear psoriasis was confirmed. Dupilumab was adjusted to 300 mg subcutaneous injection every 4 weeks. In addition, local topical treatment of fluticasone propionate cream and calcipotriol ointment was given once a day. Ten weeks later, the skin lesions gradually subsided. No recurrent rashes were seen after 6 months of follow-up.

## Discussion

Dupilumab is a fully human monoclonal antibody generated against the alpha chain of the interleukin-4 receptor, which inhibits both IL-4 and IL-13 signaling. It was approved by the FDA for the treatment of PN in September 2022 (3). Studies have demonstrated its efficacy and safety, also during COVID-19 (4–6). However, it has been linked to occurrence of paradoxical reactions, such as facial and neck dermatitis (7), urticaria (8), and lichen planus (9).

In recent years, several cases of dupilumab associated with psoriasis have been reported. Psoriasis is a well-known T cell-mediated disease characterized by increased expression of Th1 and Th17 cytokines. Since IL-4 and IL-13 are key mediators of Th2-mediated inflammation, their blockade by dupilumab may induce a shift from Th2-mediated inflammation to Th1/Th17 subsets, causing psoriasis (10). Some of the dupilumab induced psoriasis are classified as vulgaris (10), erythrodermic (11), and pustular (12) types. The recurrence of lesions following the reintroduction of dupilumab strongly supports a causal relationship. To our best knowledge, this is the first case of linear psoriasis, induced by dupilumab.

Linear psoriasis is a rare subtype of psoriasis which exhibits a linear distribution along the Blaschko’s line and mainly involves the extremities. The pathogenesis of linear psoriasis may involve a genetic mosaic phenomenon. Happle proposed that the loss of heterozygosity in somatic cells during the early stages of embryogenesis leads to somatic recombination, resulting in homozygosity for one of the genes associated with psoriasis. In this scenario, one of the daughter cells may become homozygous for a psoriasis-related gene, subsequently serving as the stem cell for a clone that proliferates in a linear fashion throughout the embryonic development of the skin, indicating that cells harboring somatic mutations linked to psoriasis migrate following the lines of Blaschko (13).

However, in order to manifest linear psoriasis, an external/environmental factor or trigger may be involved besides the genetic component. Because linear psoriasis is usually not present at birth and often occurs later in life, about one-third of patients report exogenous triggers or aggravating factors, including medications [e.g., lithium (14) and pabolizumab (15)], climate (e.g., aggravation in winter, resolution in summer), and upper digestive tract infections. Therefore, the following hypothesis was put forth: in our case, the dupilumab induced the expression of chimeric genes in linear psoriasis probably.

Currently, there are no guidelines for the treatment of linear psoriasis. It is recommended that patients with this subtype be treated following the management guidelines for psoriasis vulgaris. For the dupilumab-induced psoriasis, mild to moderate cases respond well to local treatment, including corticosteroids or vitamin D derivatives, and discontinuation of dupilumab is recommended in severe cases (16). Some reports proposed the use of Janus kinase (JAK) inhibitors as potential treatments for atopic dermatitis (AD) and psoriasis complications, such as baricitinib (17) and upadacitinib (18). The safety profile of dupilumab appears to be an important issue and, therefore, long-term monitoring is warranted. In our case, since the skin lesions were not severe, the administration of dupilumab was not terminated but rather extended the interval between injections and, prescribed topical therapy, which successfully controlled the lesions of linear psoriasis.

## Data Availability

The original contributions presented in this study are included in this article/supplementary material, further inquiries can be directed to the corresponding authors.
